# Current Opportunities and Challenges of Magnetic Resonance Spectroscopy, Positron Emission Tomography, and Mass Spectrometry Imaging for Mapping Cancer Metabolism *In Vivo*

**DOI:** 10.1155/2014/625095

**Published:** 2014-03-03

**Authors:** Gigin Lin, Yuen-Li Chung

**Affiliations:** ^1^Department of Radiology, Chang Gung Memorial Hospital at Linkou, Chang Gung University, 5 Fuhsing Street, Guishan, Taoyuan 333, Taiwan; ^2^Molecular Imaging Center, Chang Gung Memorial Hospital at Linkou, Chang Gung University, 5 Fuhsing Street, Guishan, Taoyuan 333, Taiwan; ^3^Metabolomics Core Laboratory, Chang Gung Memorial Hospital at Linkou, Chang Gung University, 5 Fuhsing Street, Guishan, Taoyuan 333, Taiwan; ^4^The Institute of Cancer Research and Royal Marsden Hospital, CRUK Cancer Imaging Centre, Downs Road, Sutton, Surrey SM2 5PT, UK

## Abstract

Cancer is known to have unique metabolic features such as Warburg effect. Current cancer therapy has moved forward from cytotoxic treatment to personalized, targeted therapies, with some that could lead to specific metabolic changes, potentially monitored by imaging methods. In this paper we addressed the important aspects to study cancer metabolism by using image techniques, focusing on opportunities and challenges of magnetic resonance spectroscopy (MRS), dynamic nuclear polarization (DNP)-MRS, positron emission tomography (PET), and mass spectrometry imaging (MSI) for mapping cancer metabolism. Finally, we highlighted the future possibilities of an integrated *in vivo* PET/MR imaging systems, together with an *in situ* MSI tissue analytical platform, may become the ultimate technologies for unraveling and understanding the molecular complexities in some aspects of cancer metabolism. Such comprehensive imaging investigations might provide information on pharmacometabolomics, biomarker discovery, and disease diagnosis, prognosis, and treatment response monitoring for clinical medicine.

## 1. Introduction

Cancer is known to have unique metabolic features [1]. Knowledge of cancer metabolism can be applied not only for early detection and diagnosis of cancer, but also in the evaluation of tumor response to medical interventions and therapies [2]. The first characterized phenotype observed in cancer cells is the Warburg effect [3], which describes a shift from energy generation through oxidative phosphorylation to energy generation through anaerobic glycolysis, even under normal oxygen concentrations. Anaerobic glycolysis produces only two ATPs per glucose and is less efficient than oxidative phosphorylation [4, 5]. Cancer cells require high-energy demand to support cell growth and proliferation; therefore cancer cells have increased glucose uptake, glycolytic activity, and lactate production and decreased mitochondrial activity, low bioenergetic status, and aberrant phospholipid metabolism [6, 7]. Several important oncogenes involved in the development and progression of common human cancers have also been found to regulate glycolysis. For example, unregulated activity of the serine/threonine kinase Akt has been shown to increase glucose uptake of tumor cells as well as increase resistance to apoptosis [8–10]. The oncogene *c-myc*, a transcription factor, controls and activates numerous glycolytic genes (e.g., hexokinase 2, enolase, and lactate dehydrogenase A) [11, 12]. Oncogenic *ras* is an important protein that controls signaling pathways for cell growth, regulation, and malignancy transformation [13] and it has been seen to increase the concentration of fructose-2,6,-bisphosphate (F2, 6BP), which is an allosteric activator of phosphofructo1-kinase, and it catalyzes the phosphorylation of fructose-6-phosphate to fructose-1,6-bisphosphate [14]. Recent advances have established further links between cancer metabolism and genetic alterations in *p53* [15], AMPK [16], PI3K [17, 18] and HIF [19].

There are growing interests in developing therapies that target important signaling pathways (e.g. PI3K [18] and MAPK [20]) and transcription factors (e.g. HIF-1 [21]) and inhibit upregulated enzymes (e.g. pyruvate dehydrogenase kinase (PDK) [22] and choline kinase [23]) and metabolite transporters (e.g. glucose transporter (Glut1) [24] and monocarboxylate transporter-1 (MCT-1) [25]). Those targeted therapies might alter cancer metabolism, and the changes in endogenous metabolites in cancer cells might be detected even before changes in tumor sizes [26–28]. Imaging methods are needed to detect early metabolic changes in cancer following treatment and these imaging readouts could be useful for monitoring the response to therapies [29, 30].

Tumor heterogeneity and its adaptations to microenvironment are important factors that could affect the effectiveness of cancer treatment; hence, the ability to image and spatially map the heterogeneity of metabolism within a tumor will be very useful for planning the treatment regime. Intratumoral heterogeneity and branched evolution are recently revealed in multiple spatially separated samples obtained from primary renal carcinomas and associated metastatic sites by using genome sequencing [31]. In addition, the metabolic heterogeneity is not only attributed to genetic alteration but is also an adaptation to hypoxic tumor microenvironment. Glycolysis confers a significant growth advantage by producing the required metabolites for cancer growth [27, 32–34], as lactate can be used by oxygenated cancer cells as oxidative fuel [35], in order to spare the glucose for the more anoxic cells in the center of the tumor [36]. This cooperation between hypoxic and normoxic tumor cells optimizes energy production and allows cells to adapt efficiently to their environmental oxygen conditions [37, 38].

Conventionally, nuclear magnetic resonance (NMR) spectroscopy [67] and mass spectrometry (MS) [68] can be used separately or in combination to provide overlapping yet complementary data to evaluate cancer [69–72]. MS have high sensitivity but the samples required prior separations using gas- or lipid-chromatography. NMR has a lower sensitivity than MS but it can measure all the detectable molecules in the sample simultaneously without the need to prior separation, cancelling out the quantification errors within the method [69]. Although analyses of biopsies with many metabolites correlated with disease aggressiveness [73], the conventional metabolomic experiments using a single biopsy of small tumor or extracting metabolites from relatively large tissue areas do not provide the spatial information of the metabolites and multiple biopsies or biopsy of normal tissue counterpart; for comparison is not feasible in routine clinical practice. Hence, noninvasive imaging would be a useful solution for spatial mapping of metabolites. The potential imaging techniques reviewed in this paper include, but are not limited to, magnetic resonance spectroscopy (MRS), dynamic nuclear polarization (DNP) MRS, positron emission tomography (PET), and mass spectrometry imaging (MSI) for tissue characterization.  Table 1 summarizes the advantages, disadvantages, and clinical applications of each imaging technique.

## 2. Magnetic Resonance Spectroscopy (MRS)

Magnetic resonance spectroscopy (MRS) is a technique that can be used in preclinical and clinical settings to study cancer metabolism [74]. It is based on nuclei such as ^1^H, ^31^P and, ^13^C that possess the property of magnetic spin. When they are placed in a magnetic field, these nuclei become aligned or opposed to the external magnetic field. Many of the nuclei are flipped into the other magnetic state when a radiofrequency pulse is applied, and the differences in the populations between these two magnetic energy states are detected as a radio wave as the system returns to equilibrium. The strength of this local field depends on the electronic environment around the nucleus. Different chemical structures possess different electronic environments and lead to nuclei resonating at slightly different frequencies. These frequencies are termed as chemical shifts, which are expressed as the dimensionless units, parts per million (ppm), in the spectrum and represent the metabolites of the measured sample [75]. Additional magnetic field gradients cause nuclei at different locations to precess at different speeds, which allows spatial information to be recovered using Fourier analysis of the measured signal [75]. By spatially encoding chemical shift information, one can generate MRS imaging by obtaining signals at different chemical shifts. This can be achieved by frequency selective radiofrequency pulses, as in stimulated echo acquisition mode (STEAM) [76] and point-resolved spectroscopy (PRESS) [77] in proton (^1^H)-MRS, or by excitation and subsequent subtraction of unwanted signals, as in image selective *in vivo* spectroscopy (ISIS) technique [78] in phosphorus (^31^P)-MRS. In addition, multivoxel spectroscopy, such as chemical shift imaging (CSI) [79], can collect spectroscopic data from multiple adjacent voxels in a single measurement.

The clinical use of spectroscopy as an adjunct to MRI has expanded dramatically over the past decades because of technical advances in hardware and pulse sequence design that have improved the spatial and temporal resolution of spectral data. Nowadays most clinical MR scanners have routine sequences for ^1^H-MRS measurements, providing a wide range of metabolic and functional information integrated with complementary MRI localization. Metabolites commonly detected in clinical ^1^H-MRS include N-acetyl-aspartate (NAA) in the normal brain tissue [39] and citrate in the normal prostate [40], and their levels decrease once being replaced by tumor. MRS detection of total choline signal has been used to diagnose and monitor breast [41], brain [42], and prostate cancers [44] and for monitoring the response to anticancer therapy [23, 72, 80]. In addition, *in vivo* ^1^H-MRS also detects signals from lipid metabolism-related compounds, such as the methylene (–CH_2_) signal at 1.3 ppm and the methyl (CH_3_) signal at 0.9 ppm [81], which originate from the fatty acyl chains of the cytoplasmic mobile lipids and not from the membrane lipids [82]. Significantly higher levels of lipid have been detected in high-grade human gliomas when compared to low-grade gliomas [81], and these changes are associated with apoptosis, necrosis, or lipid droplet formation [83–85].

^31^P-MRS could provide information on tumor bioenergetics and metabolites such as nucleoside triphosphates (NTPs), phosphocreatine (PCr), and inorganic phosphate (Pi). The production of high-energy phosphates such as NTP and PCr depends on the availability of glucose and oxygen (which are delivered to the tumors through blood vessels), and is determined by diffusion distances and local oxygen consumption rates. Therefore, in addition to blood flow parameters measured by DCE-MRI or perfusion CT, ^31^P-MRS provides an opportunity to monitor downstream biochemical reactions following reduced blood flow in hypoxic regions [85] and is useful in detecting changes in tumor reoxygenation during radiation therapy [86] as well as altered tissue pH level (measured by the Pi chemical shift changes) [87]. ^31^P-MRS also measures phospholipid metabolites, such as phosphomonoester and phosphodiester in tumor, which in turn could inform on membrane turnover and tumor response following therapies [23, 72, 81].

MRS can also directly measure the pharmacokinetics of drugs that present at relatively high concentrations in the tumor. Most *in vivo* studies on MR pharmacokinetic measurements of tumors employ fluorinated drugs, such as [5-^19^F]-fluorouracil (5-FU) and its prodrug, as detected by ^19^F MRS [88, 89], because ^19^F MRS provides relatively high sensitivity combined with low background signal. Successful image-guided delivery of a prodrug enzyme, bacterial cytosine deaminase (bCD), which converts nontoxic [5-^19^F]-fluorocytosine (5-FC) to 5-FU, was recently reported in preclinical studies [90].

Relative to conventional MRI, MRS has lower sensitivities and requires much longer acquisition times and more complex data processing, and with clinicians unfamiliar with the technique, these factors continue to limit the application of MRS in the clinical setting. Currently, there are methodologies that optimize the combined signals from multielement coil arrays to improve detection of low concentration metabolites in MRS [91], in order to improve its sensitivity and spectral resolution. In addition, the availability of higher field strength MR systems and novel techniques such as dynamic nuclear polarization hyperpolarization (DNP) can reduce some of these limitations.

## 3. DNP-MRS

**Figure 1:**
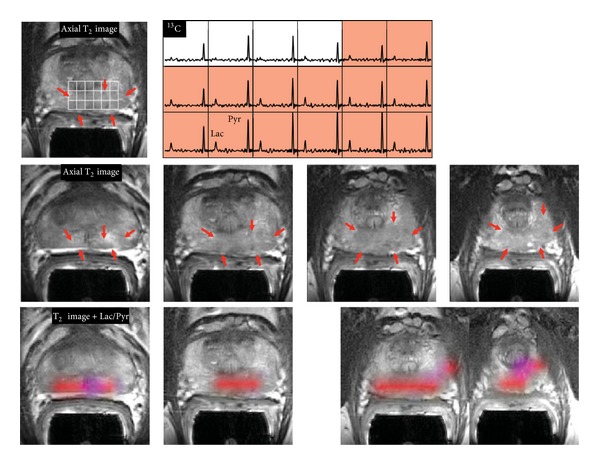
3D [1-^13^C]Pyruvate dynamic nuclear hyperpolarization magnetic resonance spectroscopy (DNP-MRS) imaging in a patient with prostate cancer. The upper panel shows an axial T2-weighted images and corresponding spectral array with the area of putative tumor highlighted by pink shading. A region of tumor was observed on the T2-weighted images (red arrows). A region of relatively high hyperpolarized [1-^13^C]lactate was observed in the same location as the abnormalities that had been observed on the multiparametric 1H staging exam. The lower panels show axial T2 images with and without metabolite overlays for different axial slices from the same patient. The colored regions in these overlays have a ratio of [1-^13^C]lactate/[1-^13^C]pyruvate ≥0.2. These demonstrated a large volume of bilateral cancer. Reprinted with permission from [106]. Copyright 2013 American Association for the Advancement of Science.

DNP is a novel imaging technique which uses specialized instrumentation to provide signal enhancements of over 10,000-folds of magnitude for stable isotope carbon-13 (^13^C) enriched compounds [92]. Simultaneous detection of multiple hyperpolarized molecules allow several metabolic pathways to be probed at the same time [93, 94], and this enhanced ^13^C signal allows the distribution of hyperpolarized ^13^C-labeled molecules within the tumor tissue to be visualized [95]. [1-^13^C]Pyruvate has been the most widely studied substrate to date because of its central role in cellular metabolism. [1-^13^C]Pyruvate also has relatively longer *T*1 relaxation time and rapid transport into the cells for subsequent metabolism [96]. Hyperpolarized [1-^13^C]pyruvate has been used to study the real-time flux of pyruvate to lactate noninvasively following anticancer therapies in xenograft models [97–101]. The first clinical trial of DNP-MRS has recently demonstrated the use of hyperpolarized [1-^13^C]pyruvate to examine prostate cancer metabolism in human [47] (Figure 1), and it paves the way to rapid translation of this exciting technology to clinical research and perhaps clinical practice [96]. Previously, the data analysis to obtain the apparent rate of pyruvate to lactate exchanges following the [1-^13^C]pyruvate DNP-MRS experiment is quite complex, as it requires the fitting of the data to a mathematical model [102]. A much simpler method to analyze this type of data has been developed recently [103], which will improve the ease of use of this methodology in studying cancer metabolism. In addition to pyruvate, extracellular pH has been measured in lymphoma xenografts by using hyperpolarized H^13^CO_3_^−^ and pH images were obtained by measuring the H^13^CO_3_^−^/^13^CO_2_ ratio in each imaging voxel [104]. [1,4-^13^C_2_]Fumarate is potentially a useful agent for detecting treatment response in tumors because the production of labeled malate was shown to be an indicator of necrotic cell death [105].

## 4. Positron Emission Tomography (PET)

Positron emission tomography (PET) is a nuclear medical imaging technique that produces three-dimensional imaging data of functional processes in the body. The system detects pairs of gamma rays emitted indirectly by positron-emitting radionuclide tracers, to provide functional or metabolic information in PET imaging [107]. When combined with X-ray computed tomography (CT), PET/CT imaging can provide both molecular information and anatomic localization. ^18^F-fluorodeoxyglucose (FDG) PET is by far the most successfully used imaging technique to study glucose uptake in tumors *in vivo.* After intravenous injection, ^18^F-FDG is transported across the cell membrane by glucose transporters and metabolized to ^18^F-FDG-6-phosphate by hexokinase [108]. In contrast to the complex metabolic fate of glucose-6-phosphate from glucose, ^18^F-FDG-6-phosphate cannot be further metabolized in the glycolytic pathway because the fluorine atom at the C_2_ position prevents ^18^F-FDG-6-phosphate from downstream catabolism. This leads to steady accumulation of ^18^F-FDG-6-phosphate in metabolically active cells such as cancer [109].

**Figure 2:**
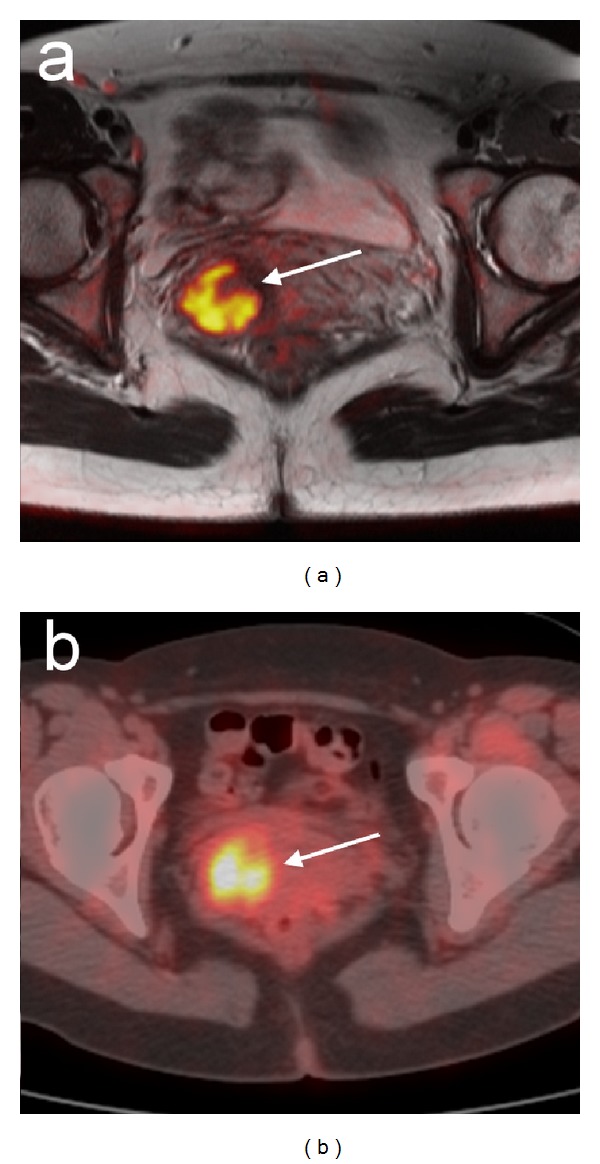
An example of PET/CT and MRI in the female pelvis. A 43-year-old female patient with a primary well-differentiated adenocarcinoma of the uterine cervix. Primary cervical tumor is highlighted (arrow) and well correlated in (a) diffusion-weighted MRI and (b) 18F-FDG PET/CT. Reprinted with permission from [56]. Copyright 2008 Springer-Verlag.

Over the past decade, ^18^F-FDG PET/CT has become a routine clinical test for staging and restaging of a variety of malignant tumors, including head and neck cancer, lymphoma, colorectal, cervical cancer (Figure 2, [56]), and many other solid organ cancers [48], with a sensitivity of about or above 90% [110]. There is considerable evidence that the reduction of ^18^F-FDG uptake is caused by a loss of viable tumor cells following chemo- and radiotherapy [111]. However, the close relationship between various oncogenic signaling pathways and tumor glucose metabolisms suggests that the drugs targeting these signal transduction pathways may have a more direct effect on cellular glucose metabolism. For example, decreased ^18^F-FDG uptakes were found in patients with gastrointestinal stromal tumors (GIST) within hours following treatment with the c-Kit inhibitor imatinib [50, 51]. Rapid reduction in ^18^F-FDG uptakes was also observed in patients with non-small cell lung cancer treated with EGFR kinase inhibitor gefitinib [52].

Although ^18^F-FDG is widely used in clinical applications, not all tumor types show a significant increase in metabolic activity on ^18^F-FDG PET imaging, for example, in prostate, neuroendocrine, and hepatic tumors [48]. Furthermore, it is difficult to evaluate malignant lesions in organs that normally take up (such as the central nervous system) or excrete FDG (such as the kidneys, urinary bladder, and prostate) or to differentiate between inflammation and cancer. Therefore, other PET tracers in addition to FDG have been developed for oncological studies [30], either for clinical use or at different stages of clinical evaluation. These compounds include ^11^C-acetate (a precursor of membrane fatty acids) in prostate cancer [112], ^11^C-methionine (a precursor of S-adenosylmethionine, which is required for polyamine synthesis) in brain tumor [113], ^18^F-choline (a substrate of choline kinase in choline metabolism) in prostate cancer [114], and ^18^F-3′-fluoro-3′-deoxy-L-thymidine (^18^F-FLT) (a substrate of thymidine kinase [TK-2] in DNA synthesis and a specific marker of cell proliferation) [115]. Efforts are also made to improve detection and measurement of low level metabolized ^18^F tracer from the ^18^F-labeled pyrimidine nucleoside analogues [116].

### 4.1. Comparison of PET and DNP-MRS

An advantage of DNP-MRS is that it does not have radiation concern that is commonly associated with PET. Although both PET and DNP-MRS can measure the uptake of labeled substrates in real-time, another key advantage of DNP-MRS is that both the injected substrate and its metabolic products can be detected and followed in real-time, allowing the observation of not only the uptake of the targeted molecule but also its downstream metabolic products [96]. In contrast, PET measures perfusion and accumulation of a tracer, but does not differentiate between metabolites containing the radionuclide or tracer per se.

The most notable limitation of DNP-MRS imaging is the very short half-life (in tens of seconds) of the hyperpolarized ^13^C-substrates, which is affected by the substrates' *T*1 value and the field strength of the MR scanner (lower field strength MR scanner improves the half-life of the hyperpolarized substrates) [96]. The hyperpolarized state decreases to its equilibrium value with a time constant according to the spin lattice relaxation time *T*1. *T*1 relaxation times are dependent on the nucleus but are also sensitive to a host of other factors including the applied field, the location in a molecule, molecular structure and motion, and the chemical environment.

In general, PET is much more sensitive than DNP-MRS [117]. PET tracers can be detected in the nano- to picomolar range [118]; whereas DNP-MRS sensitivity is still in the millimolar range. Therefore, DNP molecules are injected at concentrations that greatly exceed physiologic levels (e.g., 15–28 mmoles of pyruvate in mouse models [97, 119]), whereas PET-labeled molecules can be administered at concentrations that are unlikely to perturb normal metabolism. Although hyperpolarized [1-^13^C]pyruvate increases the sensitivity of MR imaging, signal-to-noise ratio constraints still exist for spatial and temporal resolution of ^13^C DNP-MRS, especially relative to PET, emphasizing the need for further development of MR methodology [96].

### 4.2. Potential of Simultaneous PET/MRS

The integrated PET/MRI system could offer potential in the management of cancer, with prostate, head/neck, and breast cancers among the primary indications for PET/MRI [120]. The benefit of integrating PET and MRI might not only result in improved sensitivity and spatial resolution, but also allow the specific sets of metabolic events to be examined at the same time [121]. In a preclinical murine glioma model, advancing tumor proliferation caused an increase in ^11^C-choline uptake as measured by PET, while gliosis and inflammation accounted for a high peritumoral total choline signal in MRS [122]. A decrease in ^18^F-FDG PET and changes in phosphomonoesters by ^31^P-MRS were associated with decreases in hexokinase II and Glut1 expression in HER2 expressing breast tumor xenografts and responding to trastuzumab treatment [123]. These studies exemplified that PET/MRS could be used to monitor treatment response and could provide unique information on drug biodistribution, targeting, and metabolism and serve as surrogate pharmacokinetics/pharmacodynamics (PK/PD) markers [124].

Although clinical evidence of simultaneous PET and MRS measurement is not available at present, previous reports based on the correlation of PET and MRS have demonstrated the potential usefulness of integrated PET/MRS. A significant positive correlation was found between tumor total choline concentration by ^1^H-MRS and total lesion glycolysis measured by ^18^F-FDG PET before treatment in head and neck cancer patients [125]. For primary staging in prostate cancer patients, ^1^H-MRS was reported to improve the sensitivity of ^11^C-choline PET/CT in localizing tumor in the prostate gland and achieved up to 97% of overall accuracy [126]. Combined ^1^H-MRS and DCE-MRI have improved the sensitivity of ^18^F-choline PET/CT from 62% to 92% in identifying local prostate cancer recurrence, particularly in patients with low biochemical progression after surgical treatment [127]. For breast cancer patients with an invasive ductal carcinoma of 1.5–3 cm in size, the total choline level in tumors measured by ^1^H-MRS was highly correlated with the standardized ^18^F-FDG uptake value obtained by PET/CT, and these measurements were also supported by the histologic prognostic parameters (nuclear grade, estrogen receptor status, and triple-negative lesion status) [128]. The sensitivity and specificity of total choline level by ^1^H-MRS for detecting breast cancer were 83% and 85%, respectively, and both values could be as high as 92% after technical exclusions [129].

Whether the simultaneous collection of MRS data together with PET/MRI will improve diagnosis of brain tumor remained unclear. However, evidence shows that by using choline/creatine ratio > 1.5 as a threshold, the addition of ^1^H-MRS could marginally increase the sensitivity of contrast-enhanced MRI, from 86% to 100% (*P* = .79), without altering the specificity (67%) [130]. In addition, by using cutoff points of NAA/Cho ≤ 0.61 on ^1^H-MRS and relative cerebral blood volume (rCBV) ≥ 1.50 (corresponding to diagnosis of the tumors), a sensitivity of 72% and specificity of 91% in differentiating tumors from nonneoplastic lesions have been achieved [131]. The distinction of MRS between recurrent tumor and radiation necrosis in brain tumor using the Cho/NAA ratio could be made with 85% sensitivity and 69% specificity [132].

Hepatocyte-specific (gadoxetic acid) enhanced MRI is a powerful diagnostic tool for hepatocellular carcinoma (HCC) [133], with sensitivity of about 81–90% for lesion size < 2 cm [134, 135]. For the detection of HCC, ^18^F-FDG PET/CT has a sensitivity of only around 64%–68%, which can be improved by using ^11^C-acetate [136] and ^18^F-fluorocholine [137] tracers, with reported sensitivity rising to 84% and 88%, respectively. Direct comparison of diagnostic accuracy of ^11^C-acetate or ^18^F-fluorocholine PET/CT versus hepatocyte-specific MRI on liver tumors would be of great interest; this area of research is still under investigation. Menzel et al. recently reported a multimodal *in vivo* assessment of glucose metabolism in HCC tumors using hyperpolarized [1-^13^C]pyruvate DNP-MRS and ^18^F-FDG PET [138]. The increased [1-^13^C]lactate signals in the tumor is correlated with corresponding enhanced 18F-FDG uptake. This study revealed that PET and ^13^C DNP-MRS can be used to visualize increased glycolytic flux in malignant tissue. The combined ^13^C DNP-MRS and PET readouts will allow the quantitative dissection of substrate metabolism, with respect to uptake and downstream metabolic pathways. Nonetheless, these first imaging data suggest the feasibility of 13C MRSI for future clinical use [138].

Integrated PET/MRI measurements for neuroendocrine tumors are not yet available; but efforts have been made by using somatostatin receptor-specific tracer (^68^Ga-DOTATATE) to improve lesion detection by PET [139]. ^31^P-MRShas been used to differentiate between responders and nonresponders to arterial embolization in neuroendocrine tumors, with responders that exhibit increased cell membrane renewal (higher phosphomonoester level) and energy consumption (lower NTP and higher Pi levels) [140]. For renal cell carcinoma, ^1^H-MRS can significantly differentiate tumor from healthy renal parenchyma by comparing their lipid composition [141]. An *in vitro* [1-^13^C]pyruvate DNP-MRS study of RCC cells showed a significantly higher pyruvate-to-lactate flux than the normal renal tubule cells. These metastatic RCC cells were also found to have significantly higher monocarboxylate transporter 4 (MCT4) expression and corresponding higher lactate efflux than the nonmetastatic cells, which is essential for maintaining a high rate of glycolysis [142].

## 5. Mass Spectrometry Imaging (MSI)

**Figure 3:**
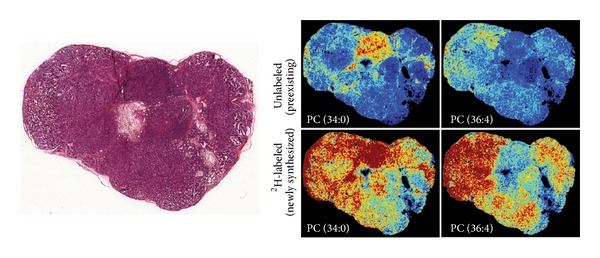
Correlation of histopathology and mass spectrometry imaging. Serial sections of the tumor are used for histopathology (left) correlation with MSI results (right). Deconvolution of spectra is performed to separate 2H-labeled and unlabeled lipids. Intensity images are generated to show the spatial distribution for both newly synthesized and preexisting lipids. Reprinted with permission from [106]. Copyright 2013 Nature Publishing Group, a division of Macmillan Publishers Limited.

Mass spectrometry imaging (MSI) is an analytical imaging technique for tissue section. It can provide a very high spatial resolution (typically 10 m) [143], but cannot provide the temporal information as the other noninvasive imaging techniques such as MRS (seconds) and PET (10 seconds to minutes). For spatial mapping, matrix-assisted laser desorption ionization-time of flight (MALDI-TOF) is the most widely used technique to analyze intact biological tissue sections in a two-dimensional fashion [143]. The matrix used in these studies is a small organic molecule with strong absorbance at the laser wavelength. They are applied on the surface of the histological section and cocrystallized with the sample. A laser pulse is used to ionize the chemical compounds on the sample and charged molecules or molecule fragments are then generated. MSI is based on the measurements of the mass-to-charge ratios, which produces spectra to determine the metabolome of the sample. This technique enables the investigation and spatial localization of both identified and unidentified molecules without any need for labeling or contrasting agents, which further facilitates the discovery of new biomarkers and their validation [144]. The damage on the biomedical tissue sections induced by laser irradiation during MALDI-MSI is relatively modest and the histological and biochemical evaluations can be performed on the same tissue slice after the MSI measurements [145] (Figure 3). The combined use of imaging modalities, such as MSI and fluorescent microscopy and histology/immunohistochemistry (IHC) [146] allows metabolic and pathological evaluations of the tissue sections in a highly precise and reliable way. MALDI MSI-based studies have been used to elucidate molecular signatures from samples with different tumor types and grades, including brain [58], oral [59], lung [60], breast [61], gastric [62], pancreatic [63], renal [64], ovarian [65], and prostate cancers [66].

MALDI-MSI is useful for metabolic imaging, albeit the average scanning time might take hours for a single mass image, depending on sample size and resolution. The target for MSI studies limits to lipid molecules of endogenous metabolites because many kinds of matrix ion peaks are observed in the low-mass range (*m*/*z* < 700), and the strong peaks that they generate interfere with the detection of the target low-molecular-weight compounds. This is because the *m*/*z* range of most lipid molecules was more than 700 and also lipids are abundant in tissues (e.g., more than 60% of the dry weight of brain tissue) and are easily ionized because of the presence of a polar head [147, 148]. MALDI-MSI was employed for imaging acylcarnitines, PC, lysophosphatidylcholine (LPC), and sphingomyelin to differentiate viable and necrotic microenvironments of breast tumor xenografts [149]. Recent breakthrough on the use of 9-aminoacridine (9-AA) as a matrix for low-molecular-weight metabolite analysis and negative mode MALDI-MS has been used to detect more than 30 metabolites (which included nucleotides, cofactors, phosphorylated sugars, amino acids, lipids, and carboxylic acids) in ischemia-reperfused rat brain tissue [150]. Hattori et al. have also reported spatiotemporal changes in energy charge, adenylates, and NADH during focal ischemia in a mouse MCAO model [151]. These findings highlight the potential applications of MSI metabolomic imaging technique to visualize spatiodynamics of some aspects of the tissue metabolome.

Although the present MALDI method is highly sensitive and well established on the MSI platform, some limitations need to be overcome before the broad range of endogenous metabolite imaging can be achieved. To date, this method can only apply to *ex vivo* tissue sections. It is generally known that, in MALDI, the detection of molecules is completely dependent on the matrix. In addition, the crystal size of the deposited matrix strongly affects both experimental reproducibility and spatial resolution in MALDI-MSI. To accelerate the use of MALDI-based metabolic imaging platform, substantial progress in matrix development and its application is required. For tissue imaging in metabolomics, nanostructure-initiator mass spectrometry (NIMS) has been investigated for spatial profiling of metabolites without the need for matrix and with reduced fragmentation [152, 153].

## 6. Concluding Remarks

The cancer metabolomics information provided by multimodality imaging techniques has revolutionized our ways of cancer treatment. Current oncologic therapy has moved forward from cytotoxic treatment to personalized therapy, such as targeting specific signal pathways or oncogene or metabolic enzymes. This would lead to altering metabolic signatures in tumor tissue, which could be monitored by using MRS or PET imaging. The nonradiation nature of MRS renders its ease of transitioning from bench to bedside. Metabolic information provided by multivoxel MRS measurements combined with the anatomical information provided by MRI can significantly improve the assessment of cancer location and extent and cancer aggressiveness. Biomarkers discovered by MRS can lead to development of new PET tracers. With the development of highly specific molecular probes, DNP-MRS and/or PET will play a major and integral role in the diagnosis, prognosis, and monitoring of treatment response in cancer and other diseases. In combination with classical histological/immunohistochemical methods, MSI analysis can provide new insights into the simultaneously occurring metabolic processes in tissue section that could not be obtained otherwise.

In the future, a combination of *in vivo* noninvasive imaging techniques (MRI anatomic imaging and functional imaging including MRS and PET) in integrated MR/PET scanners and *ex vivo* MSI validation with other tissue analytical platforms, may become the ultimate technology for unraveling and understanding some of the molecular complexities of cancer metabolism. The potential of a comprehensive study on tumor metabolism has recently been demonstrated in a glioma model, by using  ^11^C-choline PET and choline on ^1^H-MRS for *in vivo* imaging tumors, and tissue MSI for *ex vivo* validation [122]. Such combination might fulfill the function for pharmacometabolomics, biomarker discovery, disease diagnosis and prognosis, and monitoring treatment response. The development of integrated bioinformatics tools would help to handle the spatial, temporal, and multiparametric data from cancer metabolic imaging.

## Figures and Tables

**Table 1:** Comparison of major imaging techniques for studying cancer metabolism.

Imaging techniques	Advantages	Disadvantages	Clinical applications	References
Magnetic resonance spectroscopy (MRS)	(i) Widely used medical imaging technique (ii) Ability to assess multiple metabolites in one measurement (iii) No radiation concern	(i) It has relatively long acquisition time (ii) Data processing is not routine in the clinic (iii) Lack of familiarity with clinicians	Brain, head and neck, prostate, breast, and cervix	[39–46]

Dynamic nuclear polarization- (DNP-) MRS	(i) Signal enhancements of over 10,000-fold of magnitude for stable isotope carbon-13 (^13^C) enriched compounds (ii) Simultaneous detection of multiple hyperpolarized molecules allowed several metabolic pathways to be probed at the same time (iii) No radiation concern (iv) Short acquisition time (v) Real-time observation of not only the uptake of the targeted molecule but also its flux to produce downstream metabolic products	Hyperpolarized 13C-labelled substrates have very short half-life (in tens of seconds)	Prostate	[47]

Positron emission tomography (PET)	(i) Widely used in clinical applications (ii) High sensitivity	(i) Not all tumors show a significant increase in metabolic activity on FDG-PET imaging (ii) Difficult to evaluate malignant lesions in tissues that physiologically take up FDG (such as the central nervous system) or excrete FDG (such as the kidneys and bladder) or differentiate between inflammation and cancer (iii) Radiation concern (iv) It measures perfusion and accumulation of a tracer and does not differentiate between metabolites containing the radionuclide or tracer per se	Oral cancer, lymphoma, melanoma, lung cancer, esophageal cancer, and colorectal cancer Cervical Ovarian Pancreas Prostate	[48–57]

Mass spectrometry imaging (MSI)	(i) Highly sensitive (ii) It can be used to investigate both identified and unidentified molecules in spatial localized areas without any need for labeling or contrasting agents	Analytical technique of tissue section, not noninvasive imaging	Brain, oral, lung, breast, gastric, pancreatic, renal, ovarian, and prostate cancer	[58–66]
